# Breakfast Food Advertising and Prevention of Obesity: Analysis of the Nutritional Value of the Products and Discursive Strategies Used in the Breakfast Ads from 2015 to 2019

**DOI:** 10.3390/nu13010231

**Published:** 2021-01-14

**Authors:** Mireia Montaña Blasco, Mònika Jiménez-Morales

**Affiliations:** 1 Faculty of Information and Communication Sciences, Universitat Oberta de Catalunya, 08035 Barcelona, Spain; 2 Department of Communication, Pompeu Fabra University, 08018 Barcelona, Spain; monika.jimenez@upf.edu

**Keywords:** breakfast, food advertising, food policy, media, Nutri-score, nutritional value, nutrition, obesity prevention

## Abstract

Breakfast is widely considered the most important meal of the day. Despite this, the consumption of ready to eat industrial products with low nutritional value is increasing. This study correlated longitudinally the nutritional value of breakfast products with advertising discursive strategies. The research design applied quantitative analysis to compile all media advertising data from 2015 to 2019, qualitative analysis of the content, and a study of the adverts’ discourse. Moreover, a Nutri-score analysis was used to determine the products’ nutritional value. Results indicated that breakfast products advertised in Spain presented a low or very low nutritional value. In addition, they showed that the lower the nutritional value of the product, the greater the proportion of positive emotions or moods that emerge from the discourse used in the advertisement. To establish effective policies for the prevention of obesity, greater involvement of the government, and better self-regulation mechanisms for the food industry, communication agencies, and advertisers are needed. In this sense, the hedonistic language used in advertisements for unhealthy food must treated as a priority. This measure would have the aim not only of protecting minors from ads for unhealthy food that are broadcast during breakfast time but would also generate healthy eating patterns within the family.

## 1. Introduction

Breakfast has been promoted for a long time as the most important meal of the day. In Spain, only 3.36% of the population usually skips breakfast, whereas the rest of the population usually has a complete meal [1]. The Spanish complete breakfast usually consists of a dairy product, particularly milk and cereal, and especially bread and industrial bakery goods and pastries, among others.

Bread is the major contributor to total energy intake in all age groups (11.6%); this is followed by baked goods and pastries (6.8%), which ranks highest for children and adolescents and much lower for elderly adults. Meat products are the second-largest contributors (15.2%). Oils and fats (12.3%) are the third major contributor to energy intake. Milk and dairy products contributed 11.8% of total energy intake, and this is higher in children (15.9%) than in adults (11.8%). The presence of fruits and vegetables in Spanish homes during breakfast is almost imperceptible (4.7%), except for in the elderly population (8.7%) [2].

Nutritional risk factors are recognized as key drivers of obesity and other chronic diseases. In Spain, one-third of all children and two-thirds of adults suffer from excess weight, a condition that generates a direct excess medical cost of 2000 million Euros [3].

In the adult population (25–60 years), the prevalence of obesity is 17.4%, while overweight is 38.5%. Obesity is less common in women (16.7%) than in men (18.2%). Given the fact that obesity rates tend to increase with age [4], it is important to focus on the child population and its evolution over the years. According to the World Health Organization (WHO) in Spain, the prevalence of obesity among children is the highest in Europe [5].

Among the causes of this increase, several studies point to the existence of an obesogenic context, which is the result of the relationship between television (TV) viewing and high-calorie food intake [6,7,8,9,10,11]. Some of these studies have also observed that eating while watching TV is associated with a higher energy intake and an increased BMI [12,13,14,15,16]. Furthermore, although many of these products penetrate homes as children’s breakfast, the rest of the family ends up adopting these high-calorie products as part of their nutritional routine. This happens especially in those families of low and medium socioeconomic level [17].

These studies suggest that children influence the healthfulness of foods purchased when grocery shopping with their parents [18] because they tend to use nutrition heuristics rather than reading nutrition labels, and that the perceived need to move through the store quickly while shopping with children may decrease the time available to compare the nutritional benefits of products. Price, marketing, and pressure from children undermine the purpose of following a healthy diet in many homes [19]. In this sense, the pressure that the food advertising industry exerts on this group is no accident: the influence of children in the decision to buy certain products, make them end up being part of the whole family’s breakfast. Therefore the language used in the ads is not accidental either. Although these are products for the whole family, the advertising discourse tends to be especially close to the youngest members, with the aim that they end up introducing these products into the breakfast routines of the whole family.

Despite the rise of new media and communication platforms, TV continues to be the leading media in Spanish homes, reaching the highest audience percentage of the total population. According to data from the Spanish Media Research Association (AIMC, Asociación de Investigación en Medios de Comunicación) [20], 99% of children watch TV and 80% do so daily. Consequently, TV is also the channel most widely used by advertisers to market food and beverages, since it is highly effective among children and their families’ eating habits [21,22,23]. Despite this, the advertising investment for breakfast products in other media is also significant.

Children’s exposure to the marketing of unhealthy foods seems to be an unsolved problem that has a clear impact on adult health. In this sense, and considering the fact that an abundant number of unhealthy products are concentrated precisely at breakfast, this paper aims to analyze the food campaigns for all types of media from 2015 to 2019 that appealed to breakfast time, taking into consideration the nutritional quality of these advertised products and the discursive strategies used on the ads to present these products. The analysis focused on three research questions: First, what were the food campaigns that appealed to breakfast time? Second, what was the nutritional quality of these advertised products? Third, what kind of discourse was used in those campaigns?

## 2. Materials and Methods

### 2.1. Material Design and Procedure

This cross-sectional study was designed based on a mixed-methods approach. A quantitative methodology was used to compile the advertising data from 2015 to 2019 for all media. Infoadex monitors advertising activity in Spain. It has the largest database of the sector, classified with quantitative data: insertions (number of ads), occupation (size or duration of the ads), and qualitative data: creatives (different versions or executions of the same ad).

We took into consideration all types of media that Infoadex monitors: television, press, magazines, radio, outdoors, and the Internet. In this sense, a qualitative analysis was carried out based on all the food advertising campaigns that appealed to breakfast time that were captured by the Infoadex Mosaico program. The word “desayuno” (breakfast) had to be in their persuasive discourse. To that end, a total of 355 campaigns from 117 different products were taken into consideration. Advertisements, in order to perform content analysis, were based on the following proportion: television 39.15%, radio 28.17%, internet 18.03%, press 6.76%, magazines 5.07%, outdoor 2.25%, and cinema 0.56%.

### 2.2. Nutritional Analysis

To make this analysis, the different products advertised were classified considering the variable food and nutritional value of each item. The nutritional value was calculated from the Nutri-score system as in previous studies [24], which have validated its effectiveness. Nutri-score is a system for assessing the quality of processed foods that allows them to be classified into letters (A–E), each with its own color, from red to green, like a traffic light. Each product is awarded a score based on a scientific algorithm. This formula takes into account the nutrients to avoid (energy value and the number of sugars, saturated fats, and salt) and the positive ones (the amount of fiber, protein, fruit, vegetables and nuts, rapeseed oil, walnut oil, and olive oil).

The Nutri-score system was approved in Spain in 2018 and it is currently being incorporated into the packaging of food products. For those foods that did not yet have the information on their packaging, we calculated the Nutri-score label.

### 2.3. Language Analysis

Based on the transcription of the 355 ads from 117 products, we studied the lexical items that were used in their discourse. A lexical unit is understood as a word taken in one sense and supplied with all the information specifying its behavior when it is used in this sense [25].

After that, we classified these lexical items into different semantic fields and we grouped them into sets that share a common meaning, as in Fronzaroli [26]. This analysis allowed us to determine which kind of discourse was used in the ads. Finally, we analyzed the relationship between the product advertising discourse and the nutritional quality of that food.

To achieve the research objectives, we considered the following variables in the analysis of the lexical items: product, nutritional value, semantic field, and lexical items used in the ad.

## 3. Results

### 3.1. Nutritional Analysis

The results revealed that from the 355 breakfast food campaigns between 2015 and 2019, only 13.8% corresponded to products with a high nutritional value (A Food Nutritional Label). A total of 9.0% of the foods had a medium-high value (B) and 34.9% had a medium nutritional value (C), while the nutritional value of 31.8% of the advertised foods was low (D) and 10.4% was very low (E).

Concerning years, 10.53% of the products that appeared in these campaigns had a high nutritional green label (A) in 2015, 3.75% in 2016, 12.0% in 2017, 22.37% in 2018, and 20.55% in 2019. Concerning products with a medium-high nutritional score that Nutri-score classified with a light green label (B), we found that 27.63% of the foods advertised had this value in 2015, 5.00% in 2016, 0.00% in 2017, 1.32% in 2018, and 8.22% in 2019. Low and very low nutrition value products (labels D and E) increased their advertising pressure during the studied period. D and E label products campaigns represented 32.9% of the total in 2015, 36.3% in 2016, 62.0% in 2017, 39.5% in 2018 and 47.9% in 2019. The average for this period was 42.2% (Table 1).

Taking into account that some products could have more than one advertising campaign per year and that it would represent a bias, we also analyzed the Nutri-Score profile of the advertised products (Table 2). Low or very low nutrition value products (labels D and E) represented 32.0% of the total in 2015, 54.2% in 2016, 54.5% in 2017, 42.9% in 2018, and 33.4% in 2019. The average for this period was 43.6%. These data would be similar to the percentage of the previous campaign.

### 3.2. Language Analysis

To determine the use of language concerning the nutritional value of the advertised products, we carried out a linguistic study based on the message transmitted in the body of the ad. To that end, we analyzed the transcription of each ad and identified the lexical items central to each one; that is, the words that are significant or informative about the content of an ad.

Once the central lexical items of each ad had been identified, we classified them into a set of semantic fields, which showed a common core of meaningful features between these lexical items (Table 3). The creation of these semantic fields allowed us to identify the most characteristic features of the foods from the different lexical items associated with them. We must highlight that all the analyzed adverts related to meals, as they all referred to breakfast time, reinforcing the process of purchasing that product.

We observed that the vast majority of the semantic fields most commonly used make direct reference to the qualities of the foods: *con extra pepitas de chocolate* (with extra chocolate chips), *ligero* (light), *con energía* (with energy), *con L-Casei* (with Lactobacillus casei inmunitass). A total of 156 adverts can be placed in this category.

Semantic fields related to moods derived from consuming the product (e.g., being invincible, having a hunger to conquer the world, smiles, good vibes, fun, etc.) appeared in a total of 116 ads. Those related to action (e.g., family activity, to get up, to go out to enjoy, to change your breakfast…) were repeated in a total of 106 adverts. We also found a large number of ads (44) that included semantic fields related to foods themselves (e.g., biscuits, round bread, tastes…). Only one of the analyzed adverts referred to leisure time (school holidays).

Finally, we established a correlation between the nutritional value and the semantic field that described the product.

By linking these semantic fields to the products advertised, we found that 85% of cases that used the semantic field “moods” corresponded to C, D, and E Nutri-score categories (Table 4). The nutritional quality of the foods that use mainly semantic fields related to moods was low according to the Nutri-score system (e.g., chocolate powder, biscuits, cereals), very low (e.g., high-sugar biscuits, cured ham, and high-fat cheese), or practically non-existent (e.g., chocolate spread, industrial bakery products, and pastries). Additionally, 71.2% of the cases that assessed semantic fields related to the nutritional quality of the product corresponded to foods from categories C, D, and E—low (e.g., high-sugar jam, cocoa powder, industrial bakery products, and high-sugar vegetable drinks), very low (e.g., cured ham, high-fat cheese, pate, and industrial biscuits) and little or no nutritional value (e.g., butter, chocolate spread, industrial bakery products, and pastries). Regarding the foods advertised in the 44 ads in which semantic fields associated with food predominate, 63.6% belong to nutritional categories C, D, and E. Concerning the semantic field “action”, in which we found a total of 106 ads, we observed that 79.2% of the products advertised also belonged to categories C, D, and E. The only ad that used semantic fields related to “leisure time” was a product belonging to the low nutritional quality category (C label). Products from A and B categories were more related to “meals” (36.4%) and “food qualities” (28.8%) semantic fields.

## 4. Discussion

Spanish breakfast product advertising from 2015 to 2019 is dominated by foods classified as processed, with low or very low nutritional value due to their high sugar, fat, or salt content. In this sense, it should be noted that these kind of products (labels D and E), increased their advertising pressure during the studied period, year after year.

Following previous studies [27,28], there is greater exposure to food considered unhealthy than healthy eating, and most of these ads are for foods that Spanish children and part of the adult population eat for breakfast. Industrial bread, pastries, sugar-sweetened beverages, or sugary breakfast cereals, among others, are the products most frequently advertised on Spanish screens.

When relating the nutritional characteristics of these breakfast foods with the discursive analysis, one of the main findings of this study is to point out that the lower the nutritional value of the product is, the greater the presence of lexical items related to ideas linked to positive experiences. Concepts like “being invincible”, “good vibes”, “success”, “smiles” or “fun”, among others are a substantial part of the language used in the advertising of breakfast products.

As several studies show, when food ads are viewed, areas of the brain are activated that are closely related to the purchasing decision process and subsequent consumption of the product. This effect is produced especially strongly when the message derived from the ad is associated with pleasure and rewarding experiences [29].

In this sense, the World Health Organization (WHO) has already pointed out for years that this kind of advertising is “disastrously effective” especially for children because while adults know when they are being targeted by advertising, children do not, and this makes them particularly receptive and vulnerable to messages that lead to unhealthy choices that end up settling in the family breakfast [30]. The consumption of packaged food products (e.g., industrial pastries, high-sugar cereal bars, or prepared milkshakes) that do not need any kind of preparation is also increasing [31]. A direct consequence of these unhealthy habits is the low time dedicated to breakfast consumption in Spain, especially among children and adolescents, who spend between 8 and 10 min on the weekdays. This figure only increases by 3 min on the weekend [32]. It should be noted that evidence supports the suggestion that children are influenced by time spent eating meals with their families and the implementation of routine family meals appears to protect teens from engaging in negative behaviors [33].

Regarding children’s vulnerability among unhealthy food persuasive messages, some authors emphasize the relationship between emotional disorders and low nutritional value food intake. Previous studies [34,35] refer to the concept of “emotional eaters” concerning those people who seek to supplement their emotional needs through food intake. In this respect, “emotional eaters” consume more energy-dense foods with particularly low nutritional levels in response to negative emotions, compared to the rest of the population.

It can be said that in developed countries, obesity disproportionately affects individuals from lower social classes. In this sense, the relation between low-income population groups and obesity is explained by psychological distress and subsequent emotional eating as a coping strategy [29].

In this social group is where the least healthy nutritional guidelines are registered, especially at breakfast, where there are many products that, a priori, are advertised directed at children [36,37,38]. Thus, it is no coincidence that most ads for processed foods with low nutritional value base their persuasive strategy on the promise of positive moods or experiences.

People might unconsciously seek to maintain the state of happiness promised by the advertising narrative by purchasing the product. In any case, these are strategies unrelated to the product itself, which, in the case of food products, often seek to mask their nutritional deficiencies.

The qualities of the product concerning health and well-being are another of the most used arguments in the advertising discourse of breakfast products of a low or very low nutritional level. Energy properties, vitamins, or the presence of components that “enrich” the diet, predominate in the discourse of many breakfast products. This discourse is misleading to consumers who, believing in the healthy properties that the ad attributes to products with little nutritional value, end up consuming them.

This study has many strengths and also some limitations. To our knowledge, this research is the first to analyze the persuasive discourse of breakfast food advertised across all Spanish media for the last five years and to determine the nutritional quality of these products. Regarding limitations, they would be the lack of information about people’s choice of breakfast products and the research being focused on one single country. Finally, another fact to highlight is that the researchers analyzed the main internet supports, as analyzing it completely was not possible due to the complexity of the medium itself.

## 5. Conclusions

This study indicates that the breakfast products advertised in Spain presented a low or very low nutritional value. In addition, our results show that the lower the nutritional value of the product, the greater the proportion of positive emotions or moods that emerge from the discourse used in the advertisement. Given the fact that Spain leads the European ranking in terms of obesity, and that the pathology tends to increase with age, it is essential to focus on those aspects that favor an obesogenic context. As our study demonstrates, the discourse strategies used by advertising breakfast foods are especially significant in this sense.

This fact is especially harmful to children as they are unable to discern reality from fantasy and interpret the promise of getting something positive from the product as something real. For that reason, they convince their parents to buy those products of low or very-low nutritional value that end up being established in the family’s breakfast habits. Currently, in Spain, there is a regulatory framework regarding the advertising of food aimed at minors: the so-called PAOS strategy. Considering that advertised breakfast products are not just for children and that for this reason, they skip the regulatory framework, strict regulation of the advertising of unhealthy products is necessary, regardless of the consumers’ age. For this reason, we consider that in order to establish effective policies for the prevention of obesity in Spain, a greater involvement of the Spanish government, the food industry, as well as agencies and advertisers is necessary to change current advertising dynamics, focusing not only on the effects of unhealthy products advertisements on children but on the entire population. In this sense, the hedonistic language used in advertisements of unhealthy food is a priority aspect to be treated. This measure would have the aim not only of protecting minors from ads for unhealthy food but also generate healthy eating patterns within the family. Further research can explore this question by comparing results from countries with different types of regulation.

## Figures and Tables

**Table 1:** Nutri-score ^1^ classification of the advertised breakfast products campaigns by year.

Food Nutritional Label	Food Campaigns *n* (%) 2015 ^2^	Food Campaigns *n* (%) 2016 ^3^	Food Campaigns *n* (%) 2017 ^4^	Food Campaigns *n* (%) 2018 ^5^	Food Campaigns *n* (%) 2019 ^6^	Food Campaigns *n* (%) 2015–2019 ^7^
A	8 (10.5)	3 (3.8)	6 (12.0)	17 (22.4)	15 (20.3)	49 (13.8)
B	21 (27.6)	4 (5.0)	N/A	1 (1.3)	6 (8.2)	32 (9.0)
C	22 (28.9)	44 (55.0)	13 (26.0)	28 (36.8)	17 (23.3)	124 (34.9)
D	20 (26.3)	13 (16.3)	21 (42.0)	26 (34.2)	33 (45.2)	113 (31.8)
E	5 (6.6)	16 (20.0)	10 (20.0)	4 (5.3)	2 (2.7)	37 (10.4)

^1^ Nutri-score is a system for assessing the quality of processed foods that allows them to be classified into letters (A–E), each with its own color, from red to green, like a traffic light; ^2^ Total *n* = 76; ^3^ Total *n* = 80; ^4^ Total *n* = 50; ^5^ Total *n* = 76; ^6^ Total *n* = 73; ^7^ Total *n* = 355.

**Table 2:** Nutri-score classification of the advertised breakfast products by year.

Food Nutritional Label	Advertised Products *n* (%) 2015 ^1^	Advertised Products *n* (%) 2016 ^2^	Advertised Products *n* (%) 2017 ^3^	Advertised Products *n* (%) 2018 ^4^	Advertised Products *n* (%) 2019 ^5^	Advertised Products *n* (%) 2015–2019 ^6^
A	4 (16.0)	3 (12.5)	5 (22.7)	6 (21.4)	5 (27.8)	23 (19.7)
B	4 (16.0)	1 (4.2)	N/A	1 (3.6)	1 (5.6)	7 (6.0)
C	9 (36.0)	7 (29.2)	5 (22.7)	9 (32.1)	6 (33.3)	36 (30.8)
D	6 (24.0)	10 (41.7)	9 (40.9)	8 (28.6)	5 (27.8)	38 (32.5)
E	2 (8.0)	3 (12.5)	3 (13.6)	4 (14.3)	1 (5.6)	13 (11.1)

^1^ Total *n* = 25; ^2^ Total *n* = 24; ^3^ Total *n* = 22; ^4^ Total *n* = 28; ^5^ Total *n* = 18; ^6^ Total *n* = 117.

**Table 3:** Classification of the ads by semantic field.

Semantic Field	Ads *n* (%)
Meals	355 (100.0)
Food qualities	156 (43.9)
Moods	116 (32.7)
Action	106 (29.9)
Foods	44 (12.4)
Leisure time	1 (0.3)

Total *n* = 355.

**Table 4:** Correlation between the Nutri-score value and the semantic field that described the product.

Semantic Field	A Nutri-Score Advertised Products *n* (%)	B Nutri-Score Advertised Products *n* (%)	C Nutri-Score Advertised Products *n* (%)	D Nutri-Score Advertised Products *n* (%)	E Nutri-Score Advertised Products *n* (%)
Food qualities	20 (12.8)	25 (16.0)	64 (41.0)	27 (17.3)	20 (12.8)
Moods	15 (12.9)	2 (1.7)	46 (39.7)	37 (31.9)	16 (13.8)
Action	11 (10.4)	11 (10.4)	28 (26.4)	49 (46.2)	7 (6.6)
Foods	44 (22.7)	6 (13.7)	16 (36.4)	10 (22.7)	2 (4.5)
Leisure time	N/A (N/A)	N/A (N/A)	1 (100.0)	N/A (N/A)	N/A (N/A)

## Data Availability

Data sharing not applicable.
